# The effect of the Xpert MTB/RIF test on the time to MDR-TB treatment initiation in a rural setting: a cohort study in South Africa’s Eastern Cape Province

**DOI:** 10.1186/s12879-017-2200-8

**Published:** 2017-01-21

**Authors:** Joshua Iruedo, Don O’Mahony, Sikhumbuzo Mabunda, Graham Wright, Busisiwe Cawe

**Affiliations:** 1 Department of Family Medicine and Rural Health, Faculty of Health Sciences, Walter Sisulu University, Mthatha, South Africa; 2 Department of Public Health, Faculty of Health Sciences, Walter Sisulu University, Mthatha, South Africa; 3 Centre for Health Informatics Research and Development, Faculty of Health Sciences, University of Fort Hare, Alice, South Africa

**Keywords:** Xpert MTB/RIF, MDR-TB, Rural, Time-to-treatment, Cohort study

## Abstract

**Background:**

There are significant delays in initiation of multidrug-resistant tuberculosis (MDR –TB) treatment. The Xpert MTB/RIF test has been shown to reduce the time to diagnosis and treatment of MDR-TB predominantly in urban centres. This study describes the time to treatment of MDR-TB and the effect of Xpert MTB/RIF on time to treatment in a deprived rural area in South Africa.

**Methods:**

This was a retrospective cohort study analysing the medical records of patients diagnosed with MDR-TB in King Sabata Dalindyebo Sub-District between 2009 and 2014. Numerical data were reported using the Kruskal-Wallis and Wilcoxon sum rank tests and categorical data compared using the two-sample test of proportions.

**Results:**

Of the 342 patients with MDR-TB identified, 285 were eligible for analysis, of whom 145 (61.4%) were HIV positive. The median time from sputum collection to MDR-TB diagnosis was 27 days (IQR: 2–45) and differed significantly between diagnostic modalities: Xpert MTB/RIF, 1 day (IQR: 1–4; *n* = 114: *p* < 0.0001); Line Probe Assay 12 days (IQR: 8–21; *n* = 28; *p* < 0.0001); and culture/phenotypic drug sensitivity testing 45 days (IQR: 39–59; *n* = 143: *p* < 0.0001). The time from diagnosis to treatment initiation was 14 days (IQR: 8–27) and did not differ significantly between diagnostic modality. The median time from sputum collection to treatment initiation was 49 days (IQR: 20–69) but differed significantly between diagnostic modalities: Xpert MTB/RIF, 18 days (IQR: 11–27; *n* = 114; *p* < 0.0001); Line Probe Assay 29 days (IQR: 14.5–53; *n* = 28; *p* < 0.0001); and culture/phenotypic drug sensitivity, 64 days (IQR: 50–103; *n* = 143: *P* < 0.0001). Age, sex and HIV status did not influence the time intervals.

**Conclusions:**

Xpert MTB/RIF significantly reduced the time to MDR-TB treatment in a deprived rural setting as a result of a reduced time to diagnosis. However, the national target of five days was not achieved. Further research is needed to explore and address programmatic and patient-related challenges contributing to delayed treatment initiation.

**Electronic supplementary material:**

The online version of this article (doi:10.1186/s12879-017-2200-8) contains supplementary material, which is available to authorized users.

## Background

Isoniazid and Rifampicin (RIF) are still the cornerstones of Tuberculosis (TB) chemotherapy. Resistance to both these drugs defines multi-drug resistant TB (MDR-TB) [1]. While culture and in-vitro sensitivity testing is still the reference standard for MDR-TB diagnosis, newer molecular diagnostic methods significantly reduce the time to diagnosis. The World Health Organization endorsed the Line Probe Assay (LPA) in 2008 [2] followed by the Xpert MTB/RIF (Xpert) in 2010 [1]. Xpert is a cartridge based nucleic acid amplification test that can identify both Mycobacterium tuberculosis (MTB) and resistance to RIF within 2 h of specimen processing. While Xpert identifies RIF monoresistance, it is a proxy for MDR-TB [3, 4] and patients are treated as such [5]. In South Africa, diagnosis of MDR-TB was based on culture and phenotypic DST until 2008 when the Department of Health adopted LPA for smear positive sputum and culture isolates. Xpert testing started in October 2011 [6] and replaced smear microscopy as the initial test for both TB and MDR-TB diagnosis [7].

In 2014, 5.9% of all TB notifications in South Africa were MDR-TB [1]. However, only 62% of the estimated 18, 734 MDR-TB cases in 2014 were enrolled on treatment and the treatment success rate for the 2012 cohort was 52% [1]. The high loss to follow-up may be due to a high early mortality especially among HIV positive patients [8]. While the use of Xpert had not been shown to reduce mortality [9] or morbidity [10] from drug-sensitive TB, early treatment initiation in MDR-TB is associated with a reduced time to culture conversion [11, 12] and it is anticipated that early treatment will reduce transmission.

The length of time for which the patient is infectious has been implicated as a major factor in the spread of the disease [7] while treatment renders patients rapidly non-infectious [13]. This underscores the need for early diagnosis and treatment.

The time-to-treatment initiation of MDR-TB (TTTI) is defined as the period between sputum collection for drug sensitivity testing and initiation of MDR-TB treatment. The target TTTI is five days in South Africa [14]. Studies have reported significant delays in TTTI depending on the diagnostic modality. With the use of culture and phenotypic drug sensitivity testing (culture/phenotypic DST), delays of 6 to 12 weeks have been reported [15, 16]. TTTI’s for LPA are between 18 and 62 days [11, 12, 17]. The shortest TTTI’s of 8 to 17 days [18–20] are associated with Xpert, Xpert has a sensitivity of 95% and a specificity of 98% for diagnosing RIF resistance in adults [21].

The impact of Xpert on TTTI to date has been evaluated in urban settings [18–20]. The authors could not find any study evaluating the effectiveness of Xpert on TTTI in a rural setting in a developing country. The aim of this study is to describe the TTTI and the effect of the Xpert test on the TTTI among patients with MDR-TB in the King Sabata Dalindyebo (KSD) Sub-District of the Eastern Cape.

## Methods

### Definitions

**Time to diagnosis** is the time (in days) from the day of sputum collection to the day of issue of the diagnostic laboratory report of Xpert, LPA or culture/phenotypic DST to the clinic.

**Time from diagnosis to treatment** is the time taken from the day of issue of the diagnostic laboratory report to the day of initiation of MDR-TB treatment.

**Time from sputum collection to MDR-TB treatment** is the time taken from the day of sputum collection to the day of commencement of MDR-TB treatment.

This was a retrospective cohort study using medical records of patients diagnosed with MDR-TB from January 2009 until December 2014.

### Study setting

The study was conducted in the KSD Sub-District which is one of four sub-districts in the OR Tambo District Municipality. KSD has a total population of 451 710 [22] which is predominantly rural. It is served by 49 primary care facilities and three hospitals. OR Tambo Municipality is the third most deprived district in South Africa [23] with an estimated 59% of the population living below the poverty line, a high unemployment rate of 44%, no access to piped water for 51% of households, 70% living in traditional dwellings, and 30% of households having no electricity [22]. Rural residence is associated with reduced access to health services in South Africa. In a community survey, 66% of women in rural areas reported problems with access to healthcare (mainly cost, distance and transport) compared to 44% of urban women [24]. This is also worsened by the fact that 44% of South Africa’s population live in rural areas but are served by only 12% of doctors and 19% of nurses [25].

### Study population

The study population was all patients who were started on MDR-TB treatment from January 2009 to December 2014 in KSD. All patients were admitted and initiated on MDR-TB treatment in a designated TB hospital. As it was not possible to ensure that all patients with MDR-TB in KSD were identified through a review of medical records, a sample size was calculated to ensure that the number identified was sufficient for analysis.

South Africa had a MDR-TB prevalence of 5.9% (*p* = the anticipated population proportion) in 2014. Using the 95% confidence interval (z = 1.96) and a precision (d) of 3%, the minimum sample size (n) was calculated using the following equation [26]: *n* = p(100-p)z^2^/d^2^. The minimum sample size was 237 and a further 20% (~48) was added to account for lost-to-follow up or data entry errors [27]. This yielded a sample size of 285 subjects using a purposive sampling method.

### Data collection

Patients with MDR-TB from KSD were identified from EDRWeb, a national electronic drug-resistant TB treatment database with the following data elements: name, sex, age, address, HIV and antiretroviral treatment (ART), and date of treatment initiation. TB diagnostic results were obtained from the National Health Laboratory Services (NHLS), patient files and private laboratories. The date of diagnosis of MDR-TB was the date of issue to the clinic of the diagnostic laboratory report by Xpert, LPA or culture/phenotypic DST. The diagnosis of MDR-TB was made on the initial diagnostic test result after which a patient was initiated on treatment. Subsequent tests were not reported on in this study. A printout of results was obtained to ensure that the date of the first report was captured correctly except for five results where the date of issue was recorded in the patients’ files. Patients with extensively drug resistant (XDR-TB) were also included as they were initially classified as MDR-TB until the results of extended resistance testing were available.

As it was not possible to obtain the dates of sputum collection from facility registers (due to logistical challenges, including many facilities over a wide area, poor gravel roads and funding), the date of sputum registration at the laboratory was taken as a proxy for the date of sputum collection. There are daily (Monday to Friday) courier collections to transport specimens from primary care facilities to the hospital laboratories in the sub-district. All patients had access to Xpert, TB culture and LPA. Xpert was done in the two public hospitals and in the private hospital laboratories. Specimens for TB culture were sent to a centralised laboratory in KSD and those for LPA to a provincial centre in Port Elizabeth.

### Statistical analysis

Data were coded in Microsoft Excel 2010 (Microsoft Corporation, Seattle, USA) and analysed using STATA 13.1 (Stata Corp LP, College Station, Texas, USA). To analyse data that were not normally distributed, non-parametric statistics (median, interquartile range (IQR), the Wilcoxon sum rank test and the Kruskal-Wallis test) were used. Categorical data were reported using proportions and the 95% Confidence interval (95% CI) and compared using the two-sample test of proportions.

Multinomial logistic regression was used to determine predictors of “Time to Diagnosis” and “Time to Treatment Initiation”. Both instances present outputs from unadjusted models with all the variables and the adjusted model selected through the forward selection process. The model with the lowest nested Aikaike’s Information Criterion was selected as the better model. The risk ratio was used to report on the extent of associations between the predictor variables and outcome. The level of significance was set at 5% (*p*-value ≤ 0.05) and the 95% CI was also used to report on the precision of estimates.

## Results

For the years 2009–2014, 342 records were found and assessed for eligibility and exceeded the sample size calculated.

### Selection of study participants

Three hundred and forty-two patients were eligible for the study, of which 57 (17%) were excluded for the following reasons: no results were found for one patient; 22 were excluded according to the study protocol as the interval between the date of diagnosis and date of treatment initiation exceeded one year (it was considered unlikely that the decision to treat MDR-TB was based on a result obtained a year or more earlier); and 34 had ‘negative treatment start dates’ i.e. the date of treatment initiation occurred before the date of diagnosis. A total of 285 participants were included in the study.

Table 1 shows patient characteristics and demographics. There were significantly more males than females with MDR-TB and HIV. All patients had pulmonary MDR-TB. There were 279 sputum results from NHLS laboratories and six from private laboratories. The majority of participants (74.4% or *n* = 212) were between the age of 18 and 50. Comparisons of the four age categories showed no statistical difference in the proportion of participants in those age category pairs (*p* < 0.001). There was also no statistical difference between the proportion of participants who ranged from 18 to 35 years (39.3% or *n* = 112) and those who were at least 35 years old but younger than 50 (*n* = 100 or 35.7%).

**Table 1:** Patient characteristics and demographics

Sex	n (%; 95% Confidence interval)		*p*-value
Male	161 (56.5; 50.7–62.2)		
Female	124 (43.5; 37.8–49.3)		0.0019
Total	285		
AGE (Years) [category]	n (%; 95% Confidence interval)		
≤ 18 [a]	14 (4.9; 2.4–7.4)		a vs b: <0.00001
19–34 [b]	112 (39.3; 33.6–45.0)		a vs c: <0.00001
35–49 [c]	100 (35.1; 29.5–40.6)		a vs d: <0.00001
≥ 50 [d]	59 (20.7; 16.0–25.4)		b vs c: 0.298
			b vs d: <0.00001
			c vs d: 0.0001
Age (Years) by Sex	Interquartile Range	Median	
Male	29–46	37	
Female	26–48.5	34	0.1513
Total	27–48	36	
HIV	n (%; 95% Confidence interval)		
Positive	175 (61.4; 55.8–67.1)		<0.0001
Negative	110 (38.6; 32.9–44.2)		
ARV Patients	175 (61.4; 55.8–67.1)		<0.0001

**Fig. 1:**
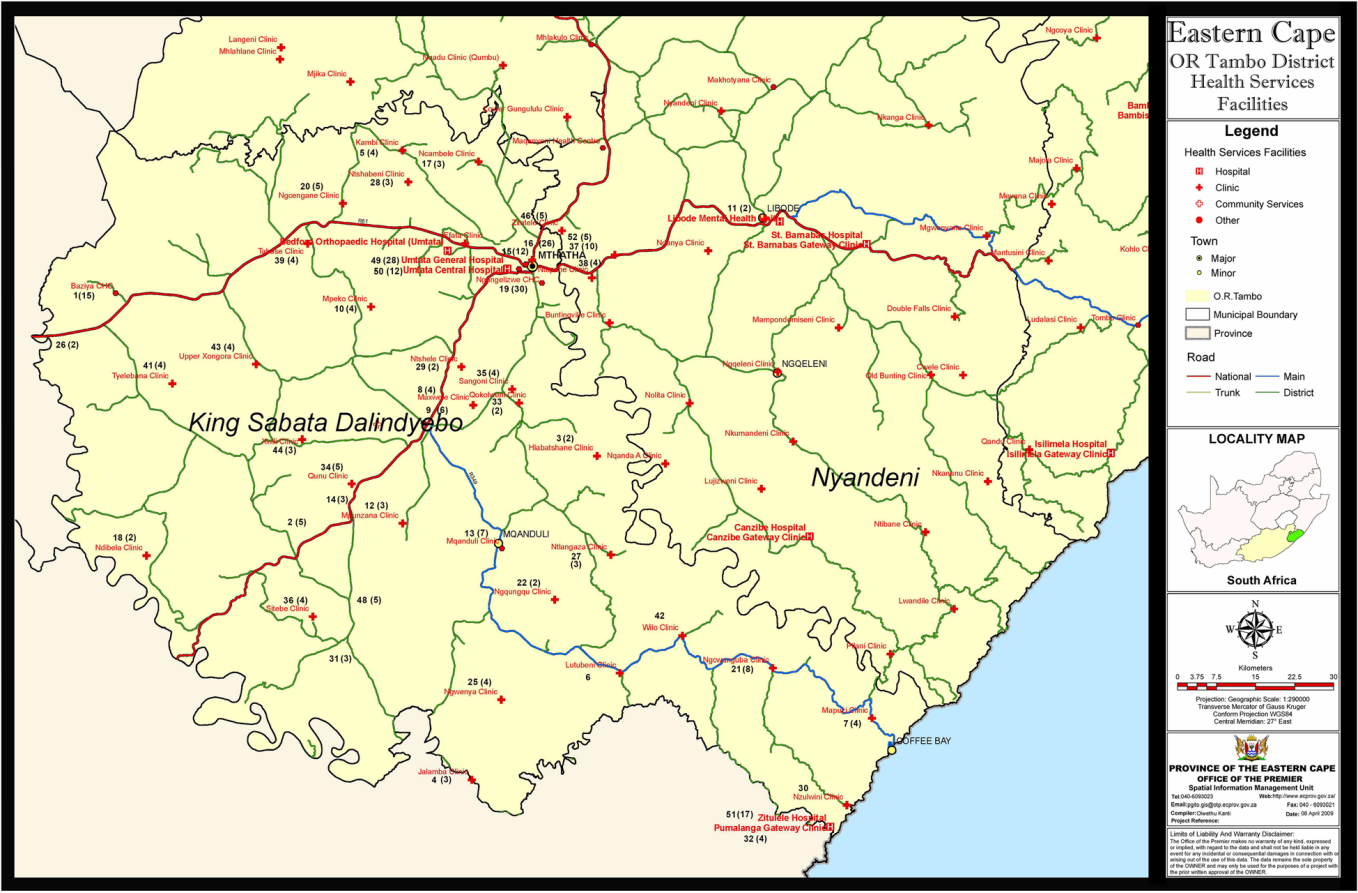
Number of patients diagnosed with MDR-TB per healthcare facility. The authors acknowledge permission from the Department of Health, Eastern Cape Province, to use and adapt the map of health facilities in the King Sabata Dalindyebo Sub-District

Figure 1 shows a map of the number of patients diagnosed with MDR-TB in each of the 52 healthcare facilities in KSD. Each facility is numbered from 1 to 52 with the number of patients with MDR-TB in brackets. The number of MDR-TB patients per facility ranged from 2 to 30 with a median of 4. Six facilities had no patient diagnosed with MDR-TB. The map shows that patients were diagnosed from all over the Sub-District. There is a marked concentration of patients around Mthatha where an academic hospital complex and a private hospital are located.

Table 2 shows that overall; the median time to diagnosis was 27 days. The time to diagnosis was shortest for Xpert (median = 1 day), was longer for LPA (median = 11.5 days) and longest for TB culture (median = 45 days). Of 28 LPA tests, 15 were done on smear positive sputum and 8 on culture isolates. Five sputum results were documented in patient files without specifying them as either a smear or a culture isolate. HIV negative patients took significantly longer to be diagnosed with MDR-TB (Median = 36-days) compared to HIV positive patients (Median = 17-days). A chi-squared test, however, showed HIV negative patients to be 15% more likely to have been diagnosed by culture as compared to being diagnosed by either Xpert or LPA but this was not statistically significant; RR = 1.150 (95% CI: 0.913–1.449; *p*-value = 0.242). In addition, there was no linear association between age and the time to diagnosis (*p* = 0.912).

**Table 2:** Time to MDR-TB diagnosis (Days)

Variable	Mode of MDR-TB diagnosis	Number	IQR	Median	*p*-value
Time to MDR TB diagnosis (Days)	Culture	143	39–59	45	
	LPA	28	8–21	11.5	0.0001
	Xpert	114	1–4	1	
	Total	285	2–45	27	
HIV status	Positive	175	2–43	17	0.0420
	Negative	110	3–51	36	
Age (Years)^a^	Co-efficient (95% CI)	R-squared			*p*-value
	0.182 (−3.06–3.43)	<0.0001			0.912

^a^A 10-year increase in age

Table 3 shows that when those diagnosed after 12 days and those diagnosed within 3–12 days are compared with those diagnosed in 2 days or less, it was found that:

**Table 3:** Predictors of the time to diagnosis (multinomial regression)

	<=2 days	3–12 days		>12 days	
	RR	RR	*p*-value	RR	*p*-value
Sex					
Female (t)	1	0.665 (0.326–1.359)	0.263	0.528 (0.300–0.931)	0.027
Female (a)	1	0.673 (0.330–1.373)	0.277	0.542 (0.309–0.950)	0.032
Age (Years)					
18 < Age < 35(t)	1	0.329 (0.056–1.953)	0.221	0.787 (0.147–4.197)	0.779
18 < Age < 35(a)	1	0.279 (0.049–1.582)	0.149	0.577 (0.112–2.977)	0.512
35 ≤ Age < 50(t)	1	0.263 (0.043–1.619)	0.150	0.845 (0.156–4.581)	0.845
35 ≤ Age < 50 (a)	1	0.218 (0.037–1.276)	0.091	0.595 (0.114–3.096)	0.538
≥ 50 (t)	1	0.305 (0.050–1.845)	0.196	0.487 (0.089–2.665)	0.407
≥ 50 (a)	1	0.293 (0.049–1.767)	0.181	0.456 (0.084–2.476)	0.363
HIV					
Negative	1	1.394 (0.621–3.128)	0.421	1.894 (0.993–3.614)	0.053

*t* total unadjusted model, *a* model adjusted for HIV status

in the unadjusted model, compared to females, males were 89.3% more likely to be diagnosed after 12 days (RR: 1.893; 95% CI: 1.074–3.336) and this was statistically significant (*p*-value: 0.027). When this association was adjusted for the HIV status the Risk Ratio marginally decreased (RR: 1.846; 95% CI: 1.053–3.238) and was also statistically significant (*p*-value: 0.032). Age was not found to predict the Time to Diagnosis.

Table 4 shows that the medians of the time it took for patients to be initiated on treatment following diagnosis were statistically similar suggesting that this time was not dependent on the type of diagnostic test used. Even when compared in pairs, all the diagnostic modalities had statistically equal medians.

**Table 4:** Time from diagnosis to treatment initiation (days)

Comparisons		Number	IQR (days)	Median	*p*-value
Time to treatment Initiation (Days)	Culture	143	8–29	14	
	LPA	28	6.5–32.5	14.5	
	Xpert	114	8–23	15	0.9521
	Total	285	8–27	14	
LPA		28	6.5–32.5	14.5	0.8294
Xpert		114	8–23	15	
LPA		28	6.5–32.5	14.5	0.7148
Culture		143	8–29	14	
Xpert		114	8–23	15	0.9616
Culture		143	8–29	14	
HIV Positive		175	7–27	14	0.5522
HIV Negative		110	8–28	15	
Age (Years)^a^	Co-efficient (95% CI)		R-squared		*p*-value
	0.035 (−3.13 3.20)		<0.0001		0.983

^a^A 10-year increase in age

There was no linear association between age of patients and the time it took for patients to start treatment. In the multinomial regression, it was found that none of the variables (age, sex, diagnostic modality, HIV status and time to diagnosis) positively predicted the time to treatment initiation.

Table 5 shows that the median time from sputum collection to MDR-TB treatment initiation was 49 days. However, when analysed by diagnostic modality, it was significantly shortest for Xpert (18 days), was longer for LPA (29 days) and longest for TB culture (64 days). When compared in pairs, all the diagnostic modalities had statistically different medians.

**Table 5:** Time from sputum collection to treatment initiation

Comparisons		Number	IQR (days)	Median	*p*-value
Time to treatment Initiation (days)	Culture	143	50–103	64	0.0001
	LPA	28	14.5–53	29	
	Xpert	114	11–27	18	
	Total	285	20–69	49	
LPA		28	14.5–53	29	0.0044
Xpert		114	11–27	18	
LPA		28	14.5–53	29	<0.0001
Culture		143	50–103	64	
Xpert		114	11–27	18	<0.0001
Culture		143	50–103	64	

### Predictors of the time from sputum collection to treatment initiation

When those initiated on treatment after 70 days and those initiated within 21–70 days were compared with those initiated in 20 days or less, it was found that: those diagnosed with LPA as opposed to those diagnosed with culture were 74.2% less likely to be initiated on treatment after 70 days as compared to being initiated on treatment on or before 20 days and this was statistically significant 0.258 (CI 0.071–0.936; *p*-value: 0.039) in the unadjusted model. Similarly, the adjusted model showed a 73.3% less likelihood 0.267 (CI 0.074–0.957; *p*-value: 0.043).

As compared to culture, those diagnosed with Xpert were 4.56 times more likely to be initiated on MDR-TB treatment between 21 and 70 days compared to being initiated after 70 days and this was statistically significant, RR = 4.560 (95% CI: 1.889–11.013; *p*-value = 0.001) in the unadjusted model. The adjusted model showed a marginal increase in the estimate where, RR = 4.563 (95% CI: 1.893–11.001; *p*-value: 0.001). Sex, HIV status and age were not associated with TTTI.

## Discussion

This study demonstrates the effectiveness (implementation under real world conditions) of Xpert in a rural setting. The results of this study show that the median TTTI was significantly reduced with use of Xpert compared to LPA and culture/phenotypic DST. The median TTTI of 18 days is similar to that of 16 days and 17 days in two studies [18, 20] while a shorter TTTI of 8 days was reported by Cox et al. [19], all conducted in urban and peri-urban settings.

The reduction in TTTI was due to a reduction in the time to diagnosis, which was significantly shorter with Xpert compared to LPA and culture/phenotypic DST. This is similar to the study by Naidoo et al. [18] where the time to diagnosis was reduced from 24 days with LPA to 1 day with Xpert; and that of Van Kampen [20] where the time to diagnosis was reduced from 75 days with culture/phenotypic DST to 1 day with Xpert. However, Naidoo et al. [18] attributed 20% of the reduction in TTTI to improved program management. Cox et al., [19] while not quantifying the contribution, also attributed their greatly improved TTTI of 8 days to both implementation of a decentralized community-based programme for drug-resistant TB, and the reduced time to diagnosis from use of Xpert.

The time from diagnosis to treatment in this study did not differ significantly between the three diagnostic modalities. Similar to this study, Hanrahan [11] and Jacobson [17] did not find a reduction in median time from diagnosis to treatment comparing LPA to culture/phenotypic DST. However, Naidoo et al. [18] reported a reduced median time from diagnosis to treatment from 14 days with LPA to 10 days with Xpert which was attributed to better programme management.

Age and sex and HIV status had no influence on TTTI, similar to the study by Naidoo et al. [18]. For time from diagnosis to treatment, age and sex and HIV status also had no influence. However, while age had no influence on time to diagnosis, males were significantly more likely to be diagnosed after 12 days compared to females. In South Africa, women utilize health care services more than men [24] and may present earlier in the course of illness. Patients with HIV were significantly likely to be diagnosed faster compared to HIV negative patients when using univariate analysis. However, multinomial regression did not show any significant association between the HIV status and time to diagnosis. As HIV positive patients typically have paucibacillary TB [28], it is expected that it would actually take longer for them to be diagnosed. The univariate analysis may have been affected by confounding factors.

In this study, the median TTTI for LPA was also significantly better compared to culture/phenotypic DST. This result was expected as LPA technology has been shown to improve TTTI mainly due to a reduction in time to diagnosis [11, 12, 17, 19]. In this study the median TTTI of 29 days for LPA was similar to that of 28 days in Khayelitsha [19]. However, the time to diagnosis of 12 days was shorter than in the comparative studies above. It is likely that this was because the majority of LPA tests were done directly on smear positive sputums and not on culture isolates. While Hanrahan et al. [11] found an overall time to diagnosis of 26 days, the time to diagnosis was only 13 days when LPA was done directly on smear positive sputums. Other studies did not report separately on the results of LPA done directly on sputum compared to culture isolates [17, 18]. However, in this study, there were few patients diagnosed with LPA. This may have been due to the limited capacity for LPA testing in the Eastern Cape Province. Furthermore, the number of LPA results was too small for a meaningful statistical analysis.

The studies from South Africa [11, 17–19], Georgia [12] and Indonesia [20] consistently show a reduction in time to diagnosis and TTTI when changing from older to newer technologies. However, neither this study nor the two published South African studies on the effectiveness of Xpert [18, 19] met the target for TTTI of five working days [14]. The challenge therefore is to reduce the time from diagnosis to treatment, which can be due to either programmatic or patient-related issues, or both.

Programmatic factors that impacted on the time from diagnosis to treatment initiation in this study included the following related to a centralized care model: all patients had to be admitted to designated hospitals approximately 200 km outside of the sub-district for initiation of treatment where they stayed until confirmed culture negative; and logistical challenges contributed to delays e.g. availability of hospital beds and transportation.

Strengthening of health systems with innovative programs of care can improve outcomes including survival, a high treatment rate (86% of those diagnosed), reduced treatment delay, and improved case detection of MDR-TB in South Africa [29, 30]. A systematic review and meta-analysis concluded that community based MDR-TB treatment programs globally were non-inferior to centralized programs [31]. The South African Department of Health is implementing a decentralised model of care [32].

The HIV prevalence (61%) in patients with MDR-TB in this study is broadly similar to studies in South Africa namely, Cape Town, 74% [19]; Cape Town, 59% [18]; Northern Cape Province, 67% [11]; and KwaZulu Natal, 71% [33]. People with HIV are at increased risk of MDR-TB. with an odds ratio of 1.24 compared to HIV negative people [34].

All patients diagnosed with HIV and MDR-TB were on antiretroviral therapy (ART). As all patients were hospitalised for initiation of MDR-TB treatment, it was easy to ensure compliance with national guidelines which state that patients with MDR-TB must be fast-tracked on ART [5, 35]. The fact that 100% of HIV positive patients were on ART means a working policy i.e. ART guidelines are well implemented. ART is associated with improved survival in patients with HIV and MDR-TB [36, 37].

In this study, there were significantly more males than females with MDR-TB and HIV with a male to female ratio of 1.4, similar to two South African studies where the ratios were 1.2 [38] and 1.3 [18]. There were also more male than female HIV negative patients with MDR-TB, with a ratio of 1.2. Farley [38] also found a higher male to female ratio of 1.7 in HIV negative patients. The male to female ratio was 1.3 among all patients diagnosed with TB in South Africa in 2014 [1]. The reasons for the male preponderance are not well characterised but may reflect biological differences in male and female susceptibility to TB infection and disease [39].

A limitation of retrospective observational studies is missing data. There are challenges with the EDRWeb system including poor-quality data entry and the lack of a unique patient identifier to track patient data [40]. In this study, missing data were managed by omitting patients from analysis (case deletion) as opposed to data imputation which is typically used with very large computerised databases [41].

Exclusion of patients with missing data from analysis may have biased results. However, there was no reason to suspect that patients with missing data differed in any respect than those who were included. The time to diagnosis and TTTI did not account for the time from collection of sputum to registration at the laboratory. If specimens arrive late in the day at the laboratory, they would be analysed the next day. Thus, the time to diagnosis and TTTI in this study may have been underestimated by approximately one day.

The strength of this study is that it analysed real world clinical practice in a deprived rural sub-district in South Africa and depicted high statistical power and reliability due to the adequate sample. It is likely that the results of this study are applicable in similar rural settings in South Africa.

## Conclusion

This study shows that Xpert reduces time to treatment initiation in a rural setting with a median TTTI of 18 days. This is similar to TTTI’s in urban settings in South Africa. However, to achieve the national target of 5 days, it is necessary to reduce the time from diagnosis to treatment by addressing health system and patient-related challenges contributing to delayed treatment initiation.

## Additional file

Additional file 1: STATA dta. Description of data: Demographics and variables. (DTA 13 kb)

## Abbreviations

ART: Antiretroviral therapy
CI: Confidence interval
culture/phenotypic DST: Culture and phenotypic drug sensitivity testing
IQR: Interquartile range
KSD: King Sabata Dalindyebo Sub-District
LPA: Line probe assay
MDR-TB: Multi-drug resistant tuberculosis
MTB: Mycobacterium tuberculosis
RIF: Rifampicin
TB: Tuberculosis
TTTI: Time-to-treatment of MDR-TB
Xpert: Xpert MTB/RIF test
